# Report of an Unusual Case

**Published:** 1865-04

**Authors:** John G. Meachem

**Affiliations:** Racine, Wisconsin


### REPORT OF AN UNUSUAL CASE.

By JOHN G. MEACHEM, M. D., of Racine, Wis.

John Rasmusen, while on a visit to Kansas, in the spring of 1859, became the subject of a severe attack of Typhoid Fever, which continued its course for a period of five weeks, and after convalescing, through careless exposure, he was again brought to his bed, and compelled to suffer three weeks more from relapse. During this second attack, he experienced more or less constant pain in the head, and was at times very delirious. He however recovered to all appearance perfectly, and so remained for several weeks. One morning he received a letter from his wife, who was then in this city, and in attempting to read it, found great difficulty. The letter was legibly written, but he could scarcely distinguish one word from another. All the symptoms of approaching amaurosis rapidly developed themselves. The air was full of floating black spots; he was unsteady in his gait; he had no confidence in stepping, for he had no appreciation of distance. In ascending a flight of stairs he would often fall forward by striking his toes against the perpendicular part of the step, instead of elevating his foot sufficiently high to place it upon the horizontal surface. His eyes looked vacant and inexpres sive; the pupils were largely dilated, and he again began to complain of cephalic pain. It was not severe, but he described it as "a pressure from within outwards." The onward march to complete amaurosis was remarkably rapid, it requiring but a few weeks to render him totally blind. He was a man possessed of more than an ordinary amount of energy and courage, and, though deprived of sight, was not content to cease his efforts to procure his own livelihood. He set about learning the art of broom making, which he succeeded in mastering in a wonderful manner, and at which business he continued until a few months prior to his decease.

In the spring of 1863, he became epileptic. His first distinct paroxysm I witnessed. It was quite severe. After it he remained unconscious for an hour. The next day he resumed his work, and, with the exception of some virtigo and a little increase of the pressure before described, he was as well as before the attack.

These epileptic paroxysms continued to recur monthly for nearly a year, after which they became more severe, with also a very marked increase of his sufferings. The pain in the head upon the right side, over the temporal bone, was complained of as being excruciatingly severe. He would often say, "I know some one is boring into my head with an auger." Not long after this character of pain began the right side of the face became paralysed, and the consequent deformity seemed more marked than usual. He was now unable to follow his usual avocation, but so determined was he to do as long as he lived, that he would send, and sometimes even go to Chicago himself, and purchase brooms by wholesale and sell them here at a little advance above the cost. Time also caused him to give this up, and he was obliged to take to his bed, which nature, however, did not require him long to keep, for, in a paroxysm of epilepsy, she relieved him forever of his sufferings. For a week before death the spine was rigidly curved forward, so that when the face was upwards the head and nates were the only bearing points. The head was also inclined to the right.

An autopsy was held twenty-four hours after death. The examination was made by Dr. J. G. Meachem, Jr., in presence of Drs. Nettleton, Wadsworth, Thompson, and myself.

On removing the aura mater, the brain presented a remarkably healthy appearance. All present remarked that they never saw a more perfect brain. A few of the pacheonian bodies were unusually developed. Two or three had embedded themselves completely in the parietal bones, and a pin could easily be pushed from their indentations through the bone. The ventricles contained more than twelve ounces of limpid serum.

Connected with the cerebellum, and lying upon the posterior surface of the central portion of the petrous portion of the temporal bone and embedded deeply in it, was a fibrous tumor of the size of a black walnut. The portion of the cerebellum giving origin to the tumor seemed a little hardened; otherwise there was no evidence whatever of disease in any part of the encephalic mass, except the effusion and an enormously enlarged foramen of Monro, which was three-quarters of an inch in diameter.

The third, as well as the lateral ventricles, was very greatly distended with serum, the pressure of which upon the optic commissare was doubtless the cause of the blindness, and the effusion must have taken place at the time, or soon after, the relapse of fever, as the result of inflammation, which was ndicated at that time by the pain in the head, delirium, etc.

It was the opinion of all the medical gentlemen present that the effusion was in no way connected with or dependent upon the presence of the tumor, as there was presented no appearance of recent inflammatory action in any part of the brain remote from the immediate vicinity of the fibrous growth. The facial paralysis was of course due to the pressure of the tumor upon the casserian ganglion, over which a portion of it lay.

